# Brief intervention for inappropriate z-hypnotics use in older adults: a before and after intervention study in primary care

**DOI:** 10.1080/02813432.2026.2660168

**Published:** 2026-04-24

**Authors:** Tahreem Ghazal Siddiqui, Tone Helene Breines Simonsen, Maria Lie Selle, Christofer Lundqvist

**Affiliations:** ^a^ Health Services Research Unit (HØKH), Akershus University Hospital, Lørenskog, Norway; ^b^ Faculty of Medicine, Institute of Clinical Medicine, University of Oslo, Oslo, Norway

**Keywords:** Hypnotics, brief intervention, older adults, randomised controlled trial, cognition, pain, sleep

## Abstract

**Background:**

Z-hypnotics are commonly prescribed for insomnia, but their use in older adults is associated with an increased risk of adverse events

**Aim:**

We examined the long-term effect of brief intervention (BI) for inappropriate z-hypnotic use, where the control group crossed over to receive the BI 6 months after baseline.

**Design and setting:**

A before-and-after intervention study was conducted in general practice. Older patients received the BI from trained general practitioners.

**Method:**

The BI group and the business-as-usual (BAU) group received the intervention with a six-month delay. The primary outcome: proportion of participants without inappropriate z-hypnotic use (≥4 weeks of use, ≥3 times per week). Secondary outcomes: the Global Sleep Assessment Questionnaire (GSAQ), pain visual analogue scale (VAS), Montreal Cognitive Assessment (MOCA), and Hospital Anxiety and Depression Scale (HADS). Patients were assessed at baseline, 6 weeks, 6 months, and 12 months.

**Results:**

We included 45 patients (31 female, mean age 69.4 years) and 21 GPs in the study. We found a significant reduction in inappropriate z-hypnotic use from baseline (68.9%) to post-treatment (27.78%), OR = 0.16, 95% CI: 0.04, 0.65, *p* = 0.01. GSAQ—insomnia score stayed low throughout the study. At 6 months, no participant reported insomnia. HADS was significantly reduced from baseline (mean 10.1) to post-intervention (mean 7.3, Cohen’s *d* = −0.44, *p* < 0.01), whereas MOCA and VAS pain did not change significantly from baseline to post-intervention.

**Conclusion:**

The proportion of patients with inappropriate z-hypnotic use decreased after BI without negatively affecting sleep, mood, pain, or cognitive function..

**Trial registration:**

clinicaltrials.gov (NCT06032715).

## Background

Among older adults (>60 years), z-hypnotics are used for insomnia and often with other central nervous system medications [1–5]. Inappropriate use of these medications is defined as drugs that pose more risks than benefits to the patients [3]. In older adults, z-hypnotic use is recommended for short term (>2–4 weeks) and at a low dose [1]. In Norway, 65% of patients with insomnia used z-hypnotics inappropriately [6,7]. Another Norwegian study found that older patients were twice as likely to receive a prescription for z-hypnotics as they were to be diagnosed with a sleep disorder [5].

The pharmacokinetics and pharmacodynamics of drugs can be influenced by ageing, thus altering drug efficiency and increasing the risk of adverse events. Older adults using z-hypnotics inappropriately (prolonged use and use of high doses) are at risk of drug-induced side effects such as delirium, decreased respiratory function, overdose, and cognitive impairment [8–11]. Patients with prolonged z-hypnotics use, and use of other sedative drugs have lower quality of life [12], experience pain and depression/anxiety symptoms [13], have increased risk of falls [14], and higher mortality compared to non-users [15,16]. Z-hypnotics users have an increased risk of dependence and substance use disorders [17,18].

We have developed and tested a brief intervention (BI) to reduce inappropriate z-hypnotics used by older adults. The BI is a communication-based intervention performed by the patient’s own physician [19]. The first part of the study was designed as a blinded randomised controlled trial (RCT) of BI *versus* a business-as-usual (BAU) arm but unfortunately became low-powered [20]. Our results showed that both the BI and control groups had a reduction in inappropriate z-hypnotic use, without a significant difference between study arms in the primary outcome. This might indicate that simply talking about sleep and medication use can help patients stop using z-hypnotics. Short interventions have previously been shown as efficient in change or reducing other anxiolytics, z-hypnotics, and sedatives in older primary care patients by informing the patients about risks, benefits of discontinuation, and withdrawal effects [21–25].

This study aims to examine the long-term effect of a BI for inappropriate z-hypnotic use in a pre-planned, blinded, delayed-start extension of our previous RCT [20], intended to increase the statistical power of the before *versus* after analysis of change in inappropriate z-hypnotic use.

## Methods

### Design

This study is part of a larger project on inappropriate z-hypnotics use in older adults in primary care in the catchment area of Akershus University Hospital in the south-east of Norway [19]. In Norway, all citizens are assigned a GP who is the main responsible physician for their medication and refers to specialised care. We therefore trained the GPs to perform the study intervention with their patients. In the present extension of our RCT [20], we conducted a before and after intervention study, where the original BAU control group also received the BI (intervention) after six months.

**Figure 1.**
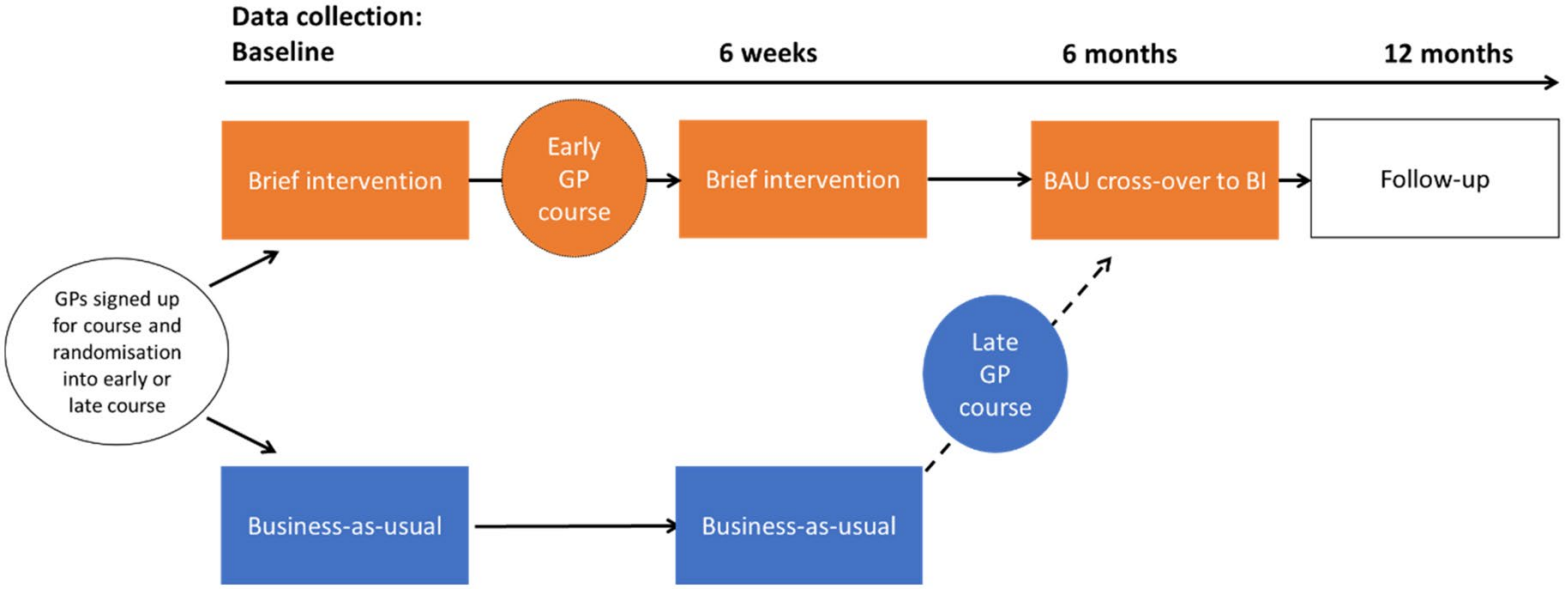
Before and after intervention study design, data collection timepoints, and GP participation, and randomisation. Screening of patients was performed after the general practitioners (GPs) had signed up for the course. GPs were cluster-randomised to early or late course. Patients of early course participants received the brief intervention directly with follow-up after 6 weeks; patients of late course participants, who were controls in the early RCT part of the study, were crossed over to also receive the intervention (BI) at 6 months. Data collection is at baseline, 6 weeks, 6 months, and 12 months follow-up after patient recruitment.

The GPs signed up for a course focusing on sleep problems and sleep medication and were cluster-randomised to receive the BI training course either early or with a 6-month delay. Their patients were screened, included at baseline, and followed for 12 months (Figure 1). In the current article, we describe the before and after intervention study, see Figure 1 (For full protocol see [19]).

### Participants

**Figure 2.**
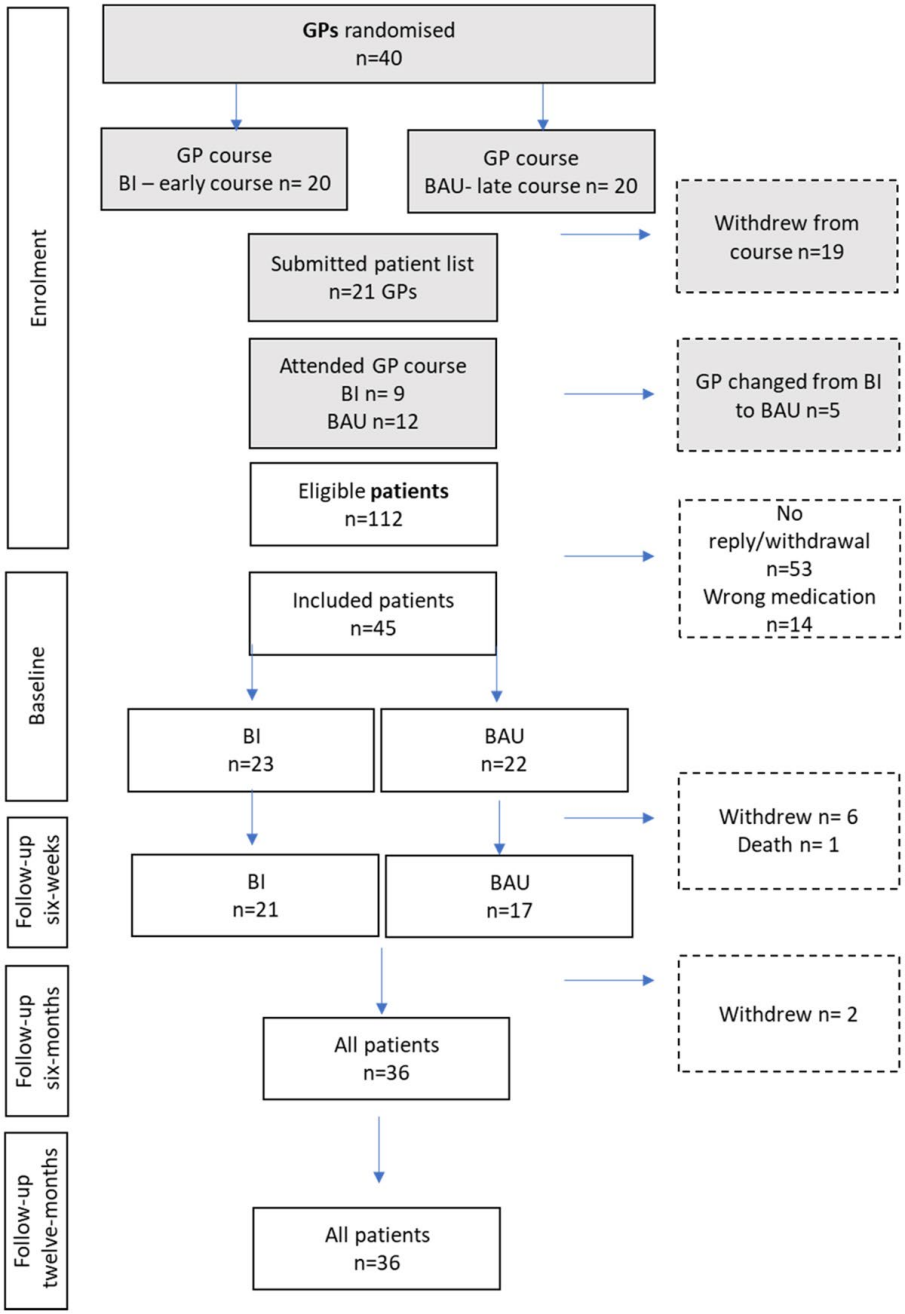
Flowchart.

GPs signed up to participate in the course and consented to provide the researchers with a patient list with names, addresses, and phone numbers of their patients aged 60 or above. We recruited the patients prospectively by contacting them through SMS using the patient contact lists. Forty GPs signed up for the course during the summer of 2023. Each GP had a median of 2 patients (range: 0–6), depending on our screening of their patients [11]. The inclusion criteria were age 60 years and above and using z-hypnotics for 4 weeks or more. Exclusion criteria were the inability to provide written consent and care home residents (as the patient’s GP then no longer has the care responsibility). Figure 2 shows the flow of participants through the study.

### Data collection

The data was collected from patients through online questionnaires at baseline, 6 weeks, 6 months, and 12 months independently of the GPs, by the researcher team, who were blinded to whether patients were in the early or late intervention group up to the 6 months’ time point.

### Measurements

#### Primary outcome

The proportion of participants with no inappropriate z-hypnotics use (≥4 weeks, ≥3 tablets per week) was compared from baseline to post intervention for the BI group (at 6 weeks) and for the delayed group, after 6 months.

#### Secondary outcomes

We assessed secondary outcomes, including sleep, pain, cognitive function, and anxiety/depression symptoms at baseline and post-intervention for the BI group (at 6 weeks) and for the delayed-intervention group (at 6 months).

#### Z-hypnotics use

We used online questionnaires to collect data on participants’ frequency and dosage of z-hypnotics use over the past 4 weeks, both at baseline and post-intervention. We also conducted phone interviews to clarify medication use for those with unclear responses. Inappropriate use was defined as using z-hypnotics for 4 weeks or more and three times per week or more; appropriate use was no z-hypnotic use or use below this threshold.

#### Demographic information

Demographic information was collected at baseline by using online questionnaires. We included questions about age (years), sex (male, female, other), and education (primary, secondary, high school, or university level).

#### Cognitive function—Montreal Cognitive Assessment

To avoid the learning effect, we sequentially used the short Norwegian versions: 7.1, 8.1, 8.2, 8.3 of the Montreal Cognitive Assessment (MOCA) to assess the global cognitive function among the participants. We used the test to detect mild cognitive impairment [26]. The test was conducted by phone interview. The short version was scored from 0 to 22; lower scores indicate cognitive impairment.

#### Pain—visual analogue scale

The questionnaire included one question related to whether patients experienced pain (yes/no). In addition, we collected answers to a visual analogue scale (VAS) (0–10 cm, higher number indicated more pain intensity) [27], and this has previously been validated in older adults [28].

#### Sleep—global sleep assessment questionnaire (GSAQ)

Global sleep assessment questionnaire (GSAQ) is an 11-item questionnaire (using, e.g. ‘never’, ‘sometimes’, ‘usually’, ‘always’), that screens for sleep disorder categories, including: insomnia, sleep apnoea, restless leg syndrome, parasomnia, and hypersomnia. In addition, secondary causes of sleep disorders, including anxiety/depression, pain, physical problems, worrying, and medication use, are recorded. We used the Norwegian version [29], and report only descriptives, number of partients labelled with ‘positive’ symptoms (item responses include ‘usually’ or ‘always’) on the given diagnoses. Negative symptoms were the remaining numbers of patients at different time points, scored as item responses ‘never’ or ‘sometimes’.

#### Anxiety and depression

The hospital anxiety and depression (HADS) scale is a 14-item scale, with a range between 0 and 21 points, divided into an anxiety score and a depression score, each with seven items, and a total score. We used the total score to examine the symptoms among the participants. Higher scores indicate increased anxiety and depression symptoms [31].

#### Relapse

We estimated the relapse rate as the number of responders (with no inappropriate use after the intervention) who again went back to inappropriate CNSD use at follow-up, either after 6 or 12 months.

### Randomisation

The statistician (MLS) received an anonymised list with ID numbers for each GP and the GP surgery to which they belonged. Since some GPs belonged to the same GP surgeries, the randomisation was based on the GP surgery clusters. This was done to avoid ‘contamination of information’ between the intervention and control arm. GPs were randomised to the early course (BI arm) or the late course (BAU control arm) (Figure 2).

### Blinding

The study coordinator (TBS) managed the data collection, and the assessors (TGS and CL) were unaware of which patients belonged to the intervention or control group at baseline and follow-up. The statistician (MLS) was blinded to which study arm the patients belonged to when analysing the data.

### Statical analyses

We used SPSS for the descriptive statistics (Version 29.0.2.0. IBM Corp. Released 2023), and the tests were performed using R version 4.4.2Relased 2024 [32]. The descriptive statistics included the primary categorical variable: z-hypnotics use, frequency of use, and secondary variable GSAQ insomnia symptoms (categorical). Secondary variables: continuous variables: MOCA, HADS, Pain VAS, and categorical variables: pain (yes/no), age, sex, and education level.

We analysed the pre-post intervention changes in the primary outcome using a mixed-effects regression model on the proportion of participants without inappropriate z-hypnotics use (≥4 weeks, ≥3 times per week). The fixed effects included treatment group (BI *vs.* delayed-intervention group), time (baseline and post-intervention; defined as 6 weeks for BI and 6 months for delayed-intervention group), interaction between the terms, age, and gender. The random part consisted of patient ID nested within GP-level. If GP-level variance was negligible, only patient ID was included. The pre-post intervention changes in the secondary outcomes (MOCA, HADS, Pain VAS) were estimated with a linear mixed-effects regression model using the same fixed and random effects as the model for the primary outcome. Model assumptions were assessed graphically. GSAQ insomnia symptoms was only described descriptively due to few insomnia observations. For the primary outcome, results are reported as odds ratios comparing baseline and post-treatment (6 weeks for BI-group and 6 months for delayed-intervention group), averaged over the two treatment groups. For continuous secondary outcomes, results are reported as Cohen’s *d* effect sizes, also averaged over the two treatment groups.

### Ethical considerations

The ethical approval was obtained from Akershus University Hospital data protection officer and the Regional Committee for Medical and Health Research Ethics (reference: 151846/556653). The participants provided written informed consent. The trial was registered on 17th August 2023 at clinicaltrials.gov (NCT06032715).

## Results

### Descriptive statistics

For the whole group, inappropriate z-hypnotics use was reduced from baseline (68.9%) to 12 months follow-up (30.6%). Daily use of z-hypnotics was decreased from 40% at baseline to 13.9% at 12-month follow-up (Table 1). Fewer participants reported pain, and anxiety/depression symptoms at 12 months compared to baseline. Only four patients reported insomnia at the first two time points, while at 6 months, no insomnia was reported, and three patients rebounded at 12 months. Sleep apnoea symptoms were the most reported, and fewer participants reported sleep apnoea post-intervention. Cognitive function was also stable from baseline (average MOCA score: 18.09) to 12-month follow-up (average MOCA score: 18.70) (Table 2).

**Table 1.** Frequency of z-hypnotics use from baseline to follow-up.

Z-hypnotics use in the last 4 weeks	Baseline (*n* = 45)	6 weeks (*n* = 21) BI group	6 months (*n* = 17) BAU group	12-months all (*n* = 36)
No use	0% (0)	28.67 (6)	35.29% (6)	33.33% (12)
Once a week	26.57% (12)	14.29% (3)	35.92% (6)	25.00% (9)
Twice a week	4.44% (2)	14.29% (3)	5.88% (1)	11.11% (4)
Three times per week	22.2% (10)	19.05 (4)	5.88% (1)	13.89% (5)
Every other day	6.67% (3)	9.52 (2)	5.88% (1)	2.78% (1)
Daily	40% (18)	14.29% (3)	11.76% (2)	13.89% (5)

Grey: not inappropriate use.

**Table 2.** Descriptive statistics for all participants from baseline to 12 months.

Variables	Baseline (*n* = 45)	6 weeks (*n* = 38)	6 months (*n* = 36)	12 months (*n* = 36)
Sex % (*n*)		Not collected	Not collected	Not collected
Female	68.9% (31)			
Male	31.1% (14)			
Age mean (*SD*)	69.40 (6.2)	Not collected	Not collected	Not collected
Education % (*n*)		Not collected	Not collected	Not collected
Primary school	20% (9)			
Secondary school	42.2% (19)			
University undergrad	22.2% (10)			
University postgrad	15.6% (7)			
Pain % (*n*)				
Yes	64.44% (29)	50.00% (19)	63.89% (23)	58.33% (21)
No	33.33% (15)	47.37% (18)	30.56% (11)	41.67% (15)
Pain VAS mean (*SD*)	3.02 (2.61)	1.70 (1.97)	3.00 (2.64)	2.64 (2.71)
MOCA mean (*SD*)	17.91(2.57)	17.94 (2.55)	18.71 (2.64)	18.70 (3.06)
HADS mean (*SD*)	10.76 (6.43)	8.62 (6.44)	7.18 (5.86)	7.36 (6.28)
Z-hypnotics % (*n*)				
No inappropriate use	31.11% (14)	63.16% (24)	72.22% (26)	69.44% (25)
Inappropriate use	68.89% (31)	36.84% (14)	27.78% (10)	30.56% (11)
GSAQ—(positive symptoms, *n*)				
Insomnia	4	4	0	3
Secondary causes of insomnia	3	1	0	2
Sleep apnoea	16	8	10	10
Hypersomnia	0	1	0	0
Restless legs syndrome	0	0	0	1
Parasomnia/shift work	0	0	0	0

GSAQ: global sleep assessment questionnaire; MOCA: Montreal Cognitive Assessment; Pain VAS: visual analogue scale; HADS: hospital anxiety and depression scale.

The sample size varies for the different time-points in the study.

### Primary outcome

Inappropriate use decreased from 68.9% at baseline to 27.8% at the 6-month follow-up. By this time, both groups had undergone the intervention; the BI-group completed 6 weeks after baseline, and the crossover group completed at 6 months. The estimated odds ratio was 0.16 [95% CI: (0.04, 0.65), *p* = 0.01].

### Secondary outcomes

HADS also improved after intervention with a significant reduction from baseline (mean 10.1, *SD* 6.3) to post-intervention (7.3, *SD* 6.0), and Cohen’s *d* of −0.44 [95% CI (−0.65, −0.23), *p* < 0.01]. MOCA did not change from baseline (mean 18.2, *SD* 2.5) to post-intervention [18.6, *SD* 2.4, Cohen’s *d* = 0.12, 95% CI (−0.24, −0.48), *p* = 0.49]. Lastly, pain VAS did not change significantly from baseline (mean 2.8, *SD* 2.4) to post-intervention [2.5, *SD* 2.6, Cohen’s *d* = −0.05, 95% CI (−0.39–0.26), *p* = 0.75].

### Post-hoc analyses of responders versus non-responders

We examined the difference in change for secondary outcomes between responders (reduced usage) *versus* non-responders (the same usage as baseline or more). We found no statistically significant differences in these outcomes.

### Relapse rates

Among the patients who were not using z-hypnotics inappropriately after the intervention and had complete data (21 patients 6 months later and 7 patients 12 months later), 2 of 21 patients had relapsed to inappropriate use 6 months later, and 1 of 7 patients relapsed between 6 and 12 months after intervention.

## Discussion

### Summary

Our findings suggest that the proportion of patients with inappropriate use decreased significantly from baseline to post-intervention. Symptoms of anxiety, depression symptoms and self-reported sleep disorders were significantly reduced after intervention. Cognitive function was stable, and self-reported pain was slightly reduced, but not significantly different from baseline to post-intervention. These findings may indicate that older adults can stop or reduce their z-hypnotics use without significant worsening of depression, anxiety, sleep problems, pain, or worsened cognitive function. Relapse rates were low across 6/12 months post-intervention.

### Comparison with existing literature

Several studies show a reduction among older patients in the use of sedatives, hypnotics, and anxiolytic medication post-interventions up to 6 months [21–25]. A 12-month trial examined the long-term effect of two short interventions (written information letters to patients or consultation with GPs) to reduce hypnotics’ use. In both groups, 45% of patients reduced their hypnotic use compared to 15% in the control group [24]. Another study showed successful reduction in benzodiazepines and z-hypnotics prescriptions after 12 months, using a GP educational workshop and monthly feedback to the GPs in the study [25]. These results are similar to our findings. The intervention methods in these studies varied; however, some form of patient and/or GP education was involved. Most of the studies used GP consultations supplemented by informational letters/instructions or booklets for patients [21–24], or educational workshops for GPs [25]. Based on our current and previous work, simple interventions for older adults in primary care may have the potential to reduce inappropriate long-term use of z-hypnotics, helping to prevent associated adverse events. Nevertheless, a recent meta-analysis suggests low certainty of evidence on the effectiveness of interventions to discontinue benzodiazepines, and additional research is needed. To increase the reduction of benzodiazepines, more research is suggested on interventions targeting patient education, medication review, and pharmacist involvement in educating physicians and patients. On the other hand, interventions with physician and patient education were less effective [33]. This is different from our findings. An explanation for this difference might be that in the BI, the GPs’ course includes information and practice on how to use the BI as a tool to change the patient’s medication-related behaviour, and involve them in shared decision making, using the patient’s own resources. This might increase the chance of reducing patients’ inappropriate use of z-hypnotics in our sample.

A meta-analysis examining the risk and benefits of z-hypnotics to improve sleep quality suggests that the numbers needed to treat for improved sleep quality are thirteen, while the numbers needed to harm from any adverse event are six, indicating a higher likelihood of experiencing harm than improved sleep quality [34]. Additionally, another meta-analysis showed minimal improvement in subjective and polysomnographic sleep latency among z-hypnotic users compared to non-users, highlighting the low clinical effect of z-hypnotics [35]. In our study, we used the GSAQ as a screening tool to examine symptoms related to sleep disorders. The GSAQ does not examine insomnia severity, sleep quality, or detect changes in sleep over time. We could have included other measures specifically examining changes in these symptoms of insomnia pre-post intervention. Insomnia is the usual indication for z-hypnotic prescription, our results show that few of the participants reported insomnia symptoms. However, sleep apnoea was the most reported problem, which was reduced after the intervention in the sample. Z-hypnotics are not recommended in older adults with sleep apnoea as this can lead to respiratory depression.

In our sample, at baseline, we found mild to moderate symptoms of anxiety and depression among the participants. However, and perhaps contrary to expectations, as insomnia treatment is often given as adjuvant treatment in anxiety and depressions, after the interventions the levels were reduced to normal, with a drop to below the cutoff of eight, which is usually taken to indicate clinically significant symptoms of anxiety and depression [30,36].

In addition, the bidirectional relationship between pain, anxiety, depression, and sleep disorders shows that pain, anxiety, and depression can lead to poor sleep, and vice versa. In a previous study, pain, anxiety, and depression symptoms were high in prolonged users of addictive medication, including z-hypnotics [13]. Moreover, sleep architecture can be altered in patients with depression with prolonged sleep latency, frequent awakenings, and increased REM sleep latency [36]. Z-hypnotics target GABA receptors to create a sedative effect that may with long-term use exacerbate depressive symptoms and anxiety, which could be an explanation for improved anxiety and depression after successful intervention.

In addition, our results show that cognitive function was within the normal range measured by the short MOCA test [26] and that cognitive function was similar post-intervention. The self-reported pain was slightly reduced (not significant) from baseline to post-intervention. Our interpretation of these findings is that older adults can stop or reduce their z-hypnotics use without significant of these symptoms. Previous research has demonstrated increased pain and decreased cognitive function in older adults using central-acting medication, including z-hypnotics [28,31]. Further research is needed to understand the underlying mechanisms for effects on sleep disorders and other, simultaneously occurring disorders.

### Strengths and limitations

The before and after intervention design of our study supplements the first RCT [20], controlled but underpowered results. The current design improved the power in comparing the intervention results between baseline and post-intervention. However, we cannot exclude that the small sample size might have affected the results. We can see an assessment effect in our study. The patients in the delayed intervention group had already changed their z-hypnotics use before the intervention. This highlights the importance of adequate non-intervention and non-assessment controls in future studies.

Our findings may not apply to all GPs in Norway, because the selected GP population was from a hospital catchment area in southeast Norway and signed up to participate in our course. Regarding the BI intervention itself, even though it is a single time point intervention, the treatment plan has options for follow-up consultations, and there could be other adaptations to individual patients, causing some of the variability in effect. Additionally, as no adjustments were made for multiple testing, there is an increased risk of type 1 error. However, under Bonferroni correction, the *p*-value for HADS would still show statistical significance, whereas the *p*-value for inappropriate use would be slightly above the adjusted significance level.

A strength of this study is that it includes a long follow-up period up to 12 months, and the design made it possible to increase the sample size also in terms of relapse follow-up. A brief systematic tool, such as BI to reduce inappropriate z-hypnotic use in older adults, can be useful to add to and implement in clinical guidelines for physicians in ordinary clinical practice. The BI is patient-centred, tailored to individual patient needs, and involves shared decision-making. The implication of this study is to enhance our understanding of z-hypnotics use related to multimorbidity, such as pain, cognition, and sleep in older adults, which are topics where current research is limited.

### Implications for practice and research

Often, patients are not aware of the negative effects on their health of prolonged z-hypnotics use, and it can be difficult to stop using z-hypnotics. We therefore developed a BI tool that clinicians can use as a support to help older adults to reduce their inappropriate z-hypnotic use. This tool is useful for assessing the severity of dependence, discussing risks and gains of quitting, and making a treatment plan and using the patient available resources to change their behaviour. Future research could focus on testing the BI tool in a larger sample and with other potentially addictive medications, such as opioids, in younger groups.

## Conclusion

The proportion of patients with inappropriate z-hypnotics use decreased significantly after intervention compared to baseline, in a time-dependent manner related to intervention time, without negative effects on sleep, cognitive function, pain, anxiety, and depression symptoms. The implementation of the BI tool in primary care practice should be further investigated. Further studies with comparison of other patient-directed or physician-directed intervention methods are also warranted.

## Supplementary Material

CONSORT.pdf

## Data Availability

Due to Norwegian regulations, potentially identifiable data may not be delivered to others. De-identified, tabulated data may be shared after a relevant written application to the authors.
